# Comparative Study of W-Shaped Angular Plate and Reconstruction Plate in Treating Posterior Wall Fractures of the Acetabulum

**DOI:** 10.1371/journal.pone.0092210

**Published:** 2014-03-14

**Authors:** Qi Zhang, Wei Chen, Xiaobo Wu, Yanling Su, Zhiyong Hou, Yingze Zhang

**Affiliations:** 1 Department of Orthopaedic Surgery, The Third Hospital of Hebei Medical University, Shijiazhuang, Hebei, P.R. China; 2 Department of Orthopaedic Surgery, The Second Hospital of Tangshan, Tangshan, Hebei, P.R. China; University of Sheffield, United Kingdom

## Abstract

**Objective:**

This study aims to assess the medium-term results of the reconstruction of posterior wall fractures using a W-shaped acetabular angular plate (WAAP) compared to those fixed using a reconstruction plate.

**Methods:**

Between July 2006 and March 2009, we performed a retrospective study, which collected data for any patient treated for a posterior acetabular wall fracture. At the time of treatment, patients were either treated using a WAAP or a pelvic reconstruction plate. The intraoperative fluoroscopic images for both groups were compared. The quality of reduction and radiological grading were assessed according to the criteria developed by Matta. The clinical assessment was based on a modified Merle d’Aubigne and Postel scoring.

**Results:**

53 patients met the inclusion criteria and were followed up for an average of 38 months. 25 patients were treated with a WAAP (study group), and 28 patients were treated with a pelvic reconstruction plate (control group). The intraoperative fluoroscopic images of the study group confirmed extra-articular screw placement in all cases. In the control group, intra-articular screw placement was observed intraoperatively in 5 patients (17.86%), and the definitive location of the periarticular hardware could not be determined in 4 patients (14.29%) during the operation. The differences between the two groups were statistically significant (*p* = 0.002). In contrast, the quality of fracture reduction, clinical outcomes, and radiological grading in the study group were not significantly different from those of the control group (*p*>0.05). The radiographic grade was strongly associated with the clinical outcomes in both the study and control groups (*p*<0.05).

**Conclusion:**

Reconstruction of posterior wall fractures of the acetabulum using a WAAP can help avoid screw penetration of the hip joint, provide a stable fixation of the posterior wall, and ensure good clinical outcomes.

## Introduction

Fractures of the posterior wall are the most common type of acetabular fractures, accounting for nearly a third of all fractures of the acetabulum [1], [2]. Despite the relative prevalence and simple configuration of fractures of the posterior wall, the outcome may be influenced by various factors. Those beyond the surgeon's control include the injury mechanism, concomitant damage to the femoral head, sciatic nerve injury, frank dislocation, and the patient's age. Critical factors that are within the surgeon’s control include the timing of surgery, surgical procedure selection, and a meticulous surgical technique. Intraarticular screw penetration remains the technical error that most influences the clinical outcome [3]. Classically, high quality intraoperative imaging is utilized to avoid screw penetration of the hip joint during surgery [4], [5]; and, 3-D navigation can improve the accuracy of screw placement [6], [7]. However, navigation technology is only used in a limited number of hospitals. Furthermore, particularly for orthopedic trauma applications, the computed tomography (CT) scanner has limited intraoperative applications [6], [7]. Another challenge of acetabular fractures is the fixation of comminuted wall fractures. Although the use of spring plates beneath a buttress plate can be an option for the fixation of comminuted posterior acetabular wall fractures, there is a risk of damage to the articular surface and dificulty of plate positioning [8]. Thus, achieving high quality of posterior wall reductions remains a challenge for orthopedic surgeons.

The purpose of this study is to assess whether the use of a W-shaped acetabular angular plate (WAAP) is helpful in avoiding screw penetration of the hip joint, while providing adequate buttressing to permit stable healing, compared to the conventional standard construct of reconstruction plates.

## Methods

A retrospective analysis of posterior wall fractures identified the fractures treated by the senior author at a single level-I trauma center from July 2006 to March 2009. Patients were considered for inclusion if they had a posterior wall fracture of the acetabulum necessitating open reduction and internal fixation (ORIF). This study consisted of any patient with a posterior acetabular fracture treated with a WAAP or pelvic reconstruction plate, during the study period. Exclusion criteria included pathological acetabular fractures, neuropathic arthropathy, dementia and other disease processes which made postoperative compliance unreliable. Patients, who were followed up for less than 24 months, were also excluded.

Preoperative radiographic imaging obtained for each patient consisted of a single standard anteroposterior pelvis film, two 45° oblique Judet views, and a 3-D CT scan. Any patient with a history of dislocation was treated preoperatively with skeletal traction, whereas all others were treated with initial bed rest. Surgery occurred as soon as the general medical condition of the patients permitted. Patients requiring ORIF were assigned alternately to either the study group or control group. A WAAP was assigned to fix the fracture of one patient, and then a reconstruction plate was assigned chronologically to treating the next one.

**Figure 1:**
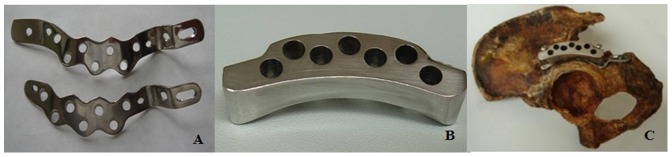
The W-shaped acetabular angular plate (A), the safe-angled drilling guild(B), and both of them assembled(C).

The Kocher-Langenbeck approach was implemented for all patients. The hip was extended, and the knee was flexed beyond 90 degrees during the retraction of the sciatic nerve. Manual distraction using a proximal femoral pin or bone hook improved the joint visualization, facilitating the removal of intraarticular loose bodies. If marginal impaction of the posterior acetabular wall was present, the impacted segment was elevated until its articular surface was flush with the articular cartilage of the femoral head and then reduced to the adjacent acetabular articular surface. A cancellous bone graft from the greater trochanter was used to fill the defect. An attempt was made to anatomically reduce any posterior wall fragments and hold them temporarily with K-wires. After the reduction, the patients in the study group were fixed using WAAP, whereas the patients in the control group were treated with a pelvic reconstruction plate. The contour and zygomorphy of the WAAP (Figure 1) matched the surface of the posterior column of the acetabulum, and the plate itself can be thought to consist of three regions: the iliac region, the danger zone region, and the ischial tuberosity region. The organization of these regions causes the plate to resemble the English alphabet letter “W”. There are two rows of drill holes in the danger zone region. A special safe-angled drilling guide was used to assist in the operation. The angles of the drilling guide were selected in congruence to the contour of the danger zone of the acetabulum, as measured using the MPR CT images[9]. The lower-surface chamber of the safe-angled drilling guide matched the contour of the danger zone aspect of the plate well, and could be assembled and disassembled easily. During the operation, the WAAP with the safe-angled drilling guide was placed along the posterior column from the ilium to the ischial tuberosity, with the outer edge of WAAP parallel to the lateral acetabular brim, and the ischial tuberosity region of the plate was placed on the ischial tuberosity. The angulation of the screw placement in the danger zone was guided by the safe-angled drilling guide. After the appropriate holes of the plate were fixed, the safe-angled drilling guide was removed. Intraoperative fluoroscopic checks, including an anteroposterior view of the hip, a obturator oblique view, an axial veiw of the screw, and a tangential view of the screw, were used to assess the fracture reduction and screw placement in each case. Dynamic fluoroscopy was not employed in the current study. Using the axial and tengential views, whether the screw violated the hip joint was determined. The determinations were made directly from the fluoroscopy monitor. If intrarticular screw penetration was recognised or highly suspected, it was screwed out, and then redrilled through the hole after adjusting the insertion angulation to fix the plate without errant screw placement into the joint.

In the control group, the wall fragments were fixed with interfragmentary screws, and the posterior column was fixed with a long 3.5 mm reconstruction plate from the ilium to ischium. The angulation of the screw placement for the holes of the plate in the danger zone was determined with caution. The intraoperative fluoroscopy at the above-mentioned views was employed frequently to help determine the periacetabular screw location as well as to assess fracture reduction.

**Figure 2:**
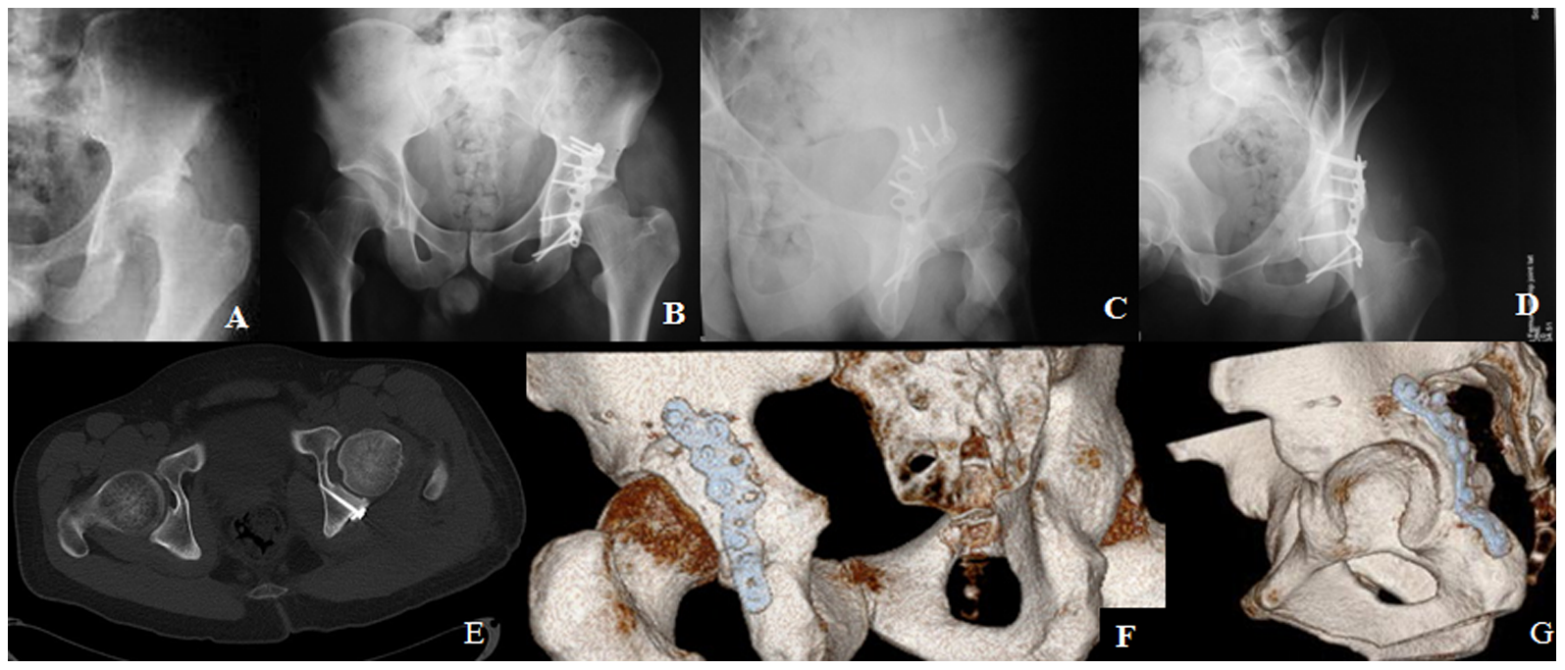
Emergency room radiograph of a 38-year-old man with a posterior wall fracture with posterior dislocation of the femoral head (A). The postoperative radiograph (B, C, D) and CT cut (E) demonstrate the extra-articular screw placement, and the VRT image shows a safe screw insertion into the posterior wall without violation into the joint (F,G).

The intraoperative fluoroscopic images, postoperative radiographs and a 3-D CT scan were utilized to assess the reduction quality and screw placement (Figure 2). The quality of reduction based on plain radiographs was assessed according to the criteria described by Matta [1]. All patients routinely received intravenous antibiotics, first administered during anesthesia induction and continuing for three days after surgery. Low molecular weight heparin was utilized for thromboprophylaxis. Prevention of heterotopic ossification was used in all patients using a 6 weeks course of Indomethacin. Patients were placed in a continuous passive-motion machine on postoperative day one. By the second postoperative day, active hip movement was encouraged, with progressive resistance of the adductors, quadriceps, and hamstrings. Similar precautions to total hip arthroplasty, such as restricting hip flexion to less than 90° and the prevention of adduction, were taught to all patients. Toe-touch weight-bearing activities with crutches or a walker were allowed for a period of 3 months following which patients were allowed to weight bear unaided.

Each patient was clinically and radiologically assessed at 1, 2, 3, 6 and 12 months, and then annually thereafter. At the final follow-up, the clinical grading was based on the modified Merle d’Aubigne and Postel scoring [1], [10]. Heterotopic ossification was graded using the classification system modified by Moed and Smith [11], and avascular necrosis of the hip was classified according to the Ficat and Arlet classification [12]. The radiological and clinical assessments were performed by two independent assessors. The assessor, who performed the clinical assessment, was also blind to the group assignment.

The data was analyzed using SPSS 13.0 (SPSS Inc., Chicago, IL, USA) by an independent biostatistician who was not directly involved with the study. The nonparametric Mann-Whitney *U*-test was used to compare the results of the intraoperative fluoroscopic images and radiographic evaluation of the reduction quality. A parametric Student’s *t*-test was used to compare the results of the operative time, blood loss and the clinical outcomes between both of the groups. The Spearman rank correlation was used to compare the relationship between clinical outcomes and associated factors.

### Ethics statement

This study has been reviewed and approved by the Institutional Review Board of the Third Hospital of Hebei Medical University. Signed informed consents were obtained from all patient. The clinical investigations have been conducted following the principles expressed in the Declaration of Helsinki.

## Results

Retrospective analysis of the patient database identified 66 patients who met the inclusion criteria, and of these, 53 patients were followed up for an average of 38 months (range, 25–60 months). The study group consisted of 25 patients treated with WAAP. The control group consisted of 28 patients treated with a pelvic reconstruction plate. There were 47 male and 6 female patients, with a mean age of 35.7 years (range, 18 to 61 years) at the time of injury. There were 31 left fractures and 22 right ones. Marginal posterior impaction of the acetabular wall was present in 25 patients. 42 patients (79.24%) had an associated posterior hip dislocation. Among those patients with a hip dislocation, 32 were reduced within 12 hours after the initial injury, 7 within 12 to 24 hours and 3 after 24 hours. All hip dislocations were treated by closed reduction. Various other concomitant injuries were found in 33 patients (62.26%): 25 had fractures of the lower extremities and 12 (22.64%) had fractures of the upper extremities requiring surgical treatment, 9 (16.98%) had head trauma, 6 (11.32%) had chest injury, 8 (15.09%) had abdominal injury that required laparotomy, and 12 (22.64%) had preoperative sciatic nerve damage. Preoperative radiographic analysis determined that there were 11 single, fragmented fractures and 42 comminuted fractures. The time from injury to operation averaged 7 days (range; 1 to 24 days). Patients in the study group were followed up for 39.40±8.56 months, and 36.75±9.43 months in the control group. The two groups of patients were comparable in sex, age, fracture severity, fractured side, preoperative sciatic nerve damage and follow-up period (Table 1, all *p*>0.05).

**Table 1:** Patient demographics of the WAAP *vs* standard plate groups.

Category	Subcategory	Study Group (n = 25)	Control group (n = 28)	*p*-value
Sex	Male	23	24	0.471
	Female	2	4	
Age (average±SD)		37.2±11.5	34.4±13.9	0.216
Fracture side	Left	16	15	0.442
	Right	9	13	
Fracture severity	Simple, fragmented fracture	3	8	0.138
	Comminuted fracture	22	20	
Hip dislocation		23	19	0.031
Preoperative sciatic nerve damage		7	5	0.378
Follow-up period (average±SD, months)		39.40±8.56	36.75±9.43	0.146

Intraoperative fluoroscopic images confirmed extra-articular screw placement in all cases in the study group. In the control group, no intra-articular screw was noted in 19 patients (67.86%), intra-articular screw placement was observed in 5 patients (17.86%), and the definitive location of the periarticular hardware could not be determined in 4 patients (14.29%). The differences between the two groups were statistically significant (Z = –3.069, *p* = 0.002). The intra-articular and highly-suspected screws in the nine patients of the control group were removed during the operation, and corrective action were taken before completion of the surgical procedure. The operative time and blood loss in the study group were 2.07±0.81 hours and 562.80±212.81 ml, respectively, which were significantly less than the operative time of 2.76±0.75 hours (t = –3.220, *p* = 0.002) and blood loss of 742.86±234.38 ml (t = –2.915, *p* = 0.005) in the control group(*p*<0.05).

The quality of fracture reduction was graded as anatomical in 19 patients (76.00%), imperfect in 4 (16.00%), and unsatisfactory in 2 (8.00%) in the study group. Among the control group patients, the reduction was graded as anatomical in 18 patients (64.29%), imperfect in 7 (25.00%) and unsatisfactory in 3 (10.71%).There was no significant difference between both groups (Z = –0.884, *p* = 0.377). The clinical outcomes are summarized in Table 2. In the study group, 76.00 % of the patients had an excellent to good outcome opposed to 60.71% in the control group. No statistically significant differences between both groups were identified (Z = –1.057, *p* = 0.291).

**Table 2:** Clinical outcomes according to the modified Merle d’Aubigne and Postel score.

Group	Excellent	good	fair	poor	total
Study Group (n (%))	8 (32.00)	11 (44.00)	3 (12.00)	3 (12.00)	25
Control group (n (%))	7 (25.00)	10 (35.71)	5 (17.86)	6 (21.43)	28

The most recent follow-up x-rays available were used for the radiological assessment according to the criteria developed by Matta. In the study group, the results were excellent in 10 (40.00%), good in 9 (36.00%), fair in 3 (12.00%) and poor in 3 (12.00%) of the patients, comparable to excellent in 9 (32.14%), good in 8 (28.57%), fair in 7 (25.00%) and poor in 4 (14.29%) of the patients in the control group. No significant difference between the two groups was identified (Z = –0.905, *p* = 0.366). Among the five patients with errant screw placement into the hip joint recognised intra-operatively, the results were excellent in 1, good in 1, fair in 2 and poor in 1, comparable to the results of the other 23 patients in the control group (*Z* = 1.123, *p* = 0.772). The Spearman rank correlation showed that the radiographic grade of the reduction was strongly associated with the clinical outcome in both the study group (r = –0.532, *p* = 0.006; Table 3) and the control group (r = –0.765, *p*< 0.000; Table 4).

**Table 3:** The clinical outcomes according to reduction quality and radiographic grade in study group.

Fracture reduction		Clinical outcome			
		Excellent (n (%))	Good (n (%))	Fair (n (%))	Poor (n (%))
Reduction quality	Anatomical (n = 19)	7 (36.84%)	9 (47.37%)	2 (10.53%)	1 (5.26%)
	Imperfect (n = 4)	1 (25.00%)	2 (50.00%)	0	1 (25.00%)
	Unsatisfactory (n = 2)	0	0	1 (50.00%)	1 (50.00%)
Radiographic grade	Excellent (n = 10)	5 (50.00%)	4 (40.00%)	1 (10.00%)	0
	Good(n = 9)	3 (33.33%)	5(55.56%)	0	1 (11.11%)
	Fair(n = 3)	0	2(66.67%)	1 (33.33%)	0
	Poor (n = 3)	0	0	1 (33.33%)	2 (66.67%)

**Table 4:** Distribution of the clinical outcomes according to reduction quality and radiographic grade in control group.

Fracture reduction		Clinical coutcome			
		Excellent (n (%))	Good (n (%))	Fair (n (%))	Poor (n (%))
Reduction quality	Anatomical (n = 18)	5 (27.78%)	9 (50.00%)	2 (11.11%)	2 (11.11%)
	Imperfect (n = 7)	2 (28.57%)	1 (14.29%)	2 (28.57%)	2 (28.57%)
	Unsatisfactory (n = 3)	0	0	1 (33.33 %)	2 (66.67%)
Radiographic grade	Excellent (n = 9)	6 (66.67%)	2 (22.22%)	0	1 (11.11%)
	Good(n = 8)	1 (12.50%)	6 (75.00%)	1 (12.50%)	0
	Fair(n = 7)	0	2 (28.57 %)	3 (42.86%)	2 (28.57%)
	Poor (n = 4)	0	0	1 (25.00%)	3 (75.00%)

Among patients in the study group, the mean functional score at the most recent follow-up was 15.64±2.56. Nine patients were found to have preoperative ipsilateral-associated peroneal nerve injuries. Heterotopic ossification developed in four patients, one of which was grade II and the other three grade I. In addition, four patients developed posttraumatic osteoarthritis, and one patient who demonstrated avascular necrosis of the femoral head and resorption of the fracture fragment exhibited poor results.

In the control group, the mean functional score was 14.75±3.17 at most recent follow-up. One patient had persistent stiffness at his knee that allowed less than 90° of further flexion, and five patients developed heterotopic ossification, two of which were grade II and the other three grade I. Four patients developed posttraumatic osteoarthritis, and avascular necrosis of the femoral head was observed in three patients. No wound infection, iatrogenic neural injury, acetabular nonunion, or pulmonary embolism were noted in either group.

## Discussion

A high percentage of long-term satisfactory outcomes can be expected following the anatomic reduction and internal fixation of fractures of the posterior wall [13], [14]. Advanced surgical techniques are often required to achieve excellent clinical outcomes, however, especially in the presence of a comminuted fracture [15]. Among posterior acetabular fractures treated operatively, the presence of intraarticular hardware is a serious complication, and has been reported in up to 4% of patients [16]–[18]. Intraarticular hardware may lead to joint destruction [3], and as such, careful screw insertion and intraoperative fluoroscopy may be necessary to ensure a safe implant location during surgery. It is possible that improvements in the fracture fixation equipment might allow for decreased fluoroscopy time in the operating room, and potentially decreased operative time overall. The uniquely angled design of the drilling guide removes the necessity of evaluating complications, thus facilitating the operation. In the control group, intraoperative fluoroscopy was used frequently in determining the periacetabular screw location, particularly for the axial and tangential views of the screws. Good-quality pelvic fluoroscopy was not always easy to achieve, however, particularly in obese patients. Therefore, the total operative time and blood loss in the control group was more than that observed in the study group. The avoidance of screw placement in the danger zone of the pelvis could minimize the possibility of screw penetration into the hip joint [19]. However, by not placing a screw in the danger zone, the overall stiffness of the internal fixation would be reduced, potentially leading to a loss of fixation in time. According to our experience and the present results, ORIF using WAAP is effective in avoiding screw penetration of the hip joint, especially in the danger zone, thus reducing the operative duration and blood loss.

A variety of methods have been described for posterior acetabular wall fracture fixation. Reconstruction plates, locking reconstruction plates, and buttressing the posterior wall in conjunction with lag screws or spring plates are among the most commonly employed techniques [20]. Results with these methods have not always been good, however, and the presence of comminution of the posterior wall remains one of the most important reasons for this. Failure of fixation of posterior wall fractures is a devastating complication that is best prevented by the provision of rigid stability intraoperatively. Inadequate fixation of the fracture - especially in the presence of the high mechanical demands of the hip joint - can lead to a catastrophic loss of fixation, which can in turn result in hip joint instability. Loss of fixation almost inevitably leads to a poor result, and therefore augmentation of the fixation of the posterior wall with some methods has been advocated [21], [22]. Ebraheim et al. [21] reported that excellent to good results were achieved in 24 of 32 comminuted posterior wall fractures treated by the buttress technique (fixation with spring plates and reconstruction plates). However, care must be taken in positioning spring plates at the posterior wall, as placement of the plate's teeth into the labrum risks damage to the femoral head cartilage [23]. Comminuted or very peripheral posterior wall fragments also pose a challenge, as it is difficult to reduce and fix every fragment with lag screws, as well as to position the buttress plate effectively in that setting. The “two-level reconstruction” technique for comminuted posterior wall fractures has been advocated by Giannoudis et al. [24]. However, additional operative time is required to ensure extra-articular subchondral screw placement by fluoroscopy prior to definitive fixation of the overlying cortical fragment. Another potential pitfall with this fixation technique is that removal of the subchondral screws is impossible in the setting of postoperative infection or joint erosion. Olson et al. [22] has reported on the use of calcium phosphate to fill incongruous fracture reductions, concluding that it may restore the loading characteristics to near-normal parameters. Further biomechanical and in vivo analyses of this practice are required however, before it can be applied clinically.

The main indication for using WAAP is in the presence of comminution of the posterior wall. In the current study, for those patients who had severe comminution with more than three major fragments, the new device functioned as a splint and provided rigid fixation to the fracture site. 76.00% of the patients had an excellent to good outcome, as opposed to 60.71% in the control group, even though a statistical significance was not determined because of the small sample size. All patients treated using WAAP achieved a congruent reduction, rigid fracture fixation, and sound union within 3 months. Therefore, it is believed that this device can provide a stable fixation of the posterior wall that is amenable to an early range of motion and weight bearing. No loss of reduction, non-union, or residual instability were noted at the final follow-up in any patient.

Limitations to this study include the small sample size and relatively short duration of the follow ups. A correlation between the clinical and radiological outcome and the WAAP could not be determined partially due to the small sample size. Moving forward, long term data with a larger sample size is necessary, and such clinical outcome data will be recorded and presented in the future. Besides, the method of patient allocation is “alternate allocation”, which is open to the treating surgeons and has limitations and potential biases. This alllocation method may influence the decision to recruit a patient to ORIF or not. A randomized controlled trial will be required to assess the clinical outcomes of the reconstruction of posterior wall fractures using a WAAP or a reconstruction plate more objectively.

## Conclusions

Reconstruction of posterior wall fractures of the acetabulum, in particular comminuted fractures, using WAAP has been demonstrated to produce good results. This device helps to avoid intraarticular screw penetration and reduce operative duration and blood loss. The device further provides a stable fixation of the posterior wall that is amenable to early range of motion and weight bearing postoperatively, and results in a good clinical outcome.
